# Enhancing osteogenic differentiation of dental pulp stem cells through rosuvastatin loaded niosomes optimized by Box-Behnken design and modified by hyaluronan: a novel strategy for improved efficiency

**DOI:** 10.1186/s13036-024-00406-7

**Published:** 2024-01-26

**Authors:** Zaynab Sadeghi Ghadi, Amin Asadi, Younes Pilehvar, Mozhgan Abasi, Pedram Ebrahimnejad

**Affiliations:** 1 Department of Medical Nanotechnology, School of Advanced Technologies in Medicine, Mazandaran University of Medical Sciences, Sari, Iran; 2 The Health of Plant and Livestock Products Research Center, Mazandaran University of Medical Sciences, Sari, Iran; 3 Student Research Committee, Faculty of Pharmacy, Mazandaran University of Medical Sciences, Sari, Iran; 4 Pharmaceutical Sciences Research Center, Hemoglobinopathy Institute, Mazandaran University of Medical Sciences, Sari, Iran; 5 Cellular and Molecular Research Center, Cellular and Molecular Medicine Research Institute, Urmia University of Medical Sciences, Urmia, Iran; 6 Immunogenetics Research Center, Department of Tissue Engineering and Applied Cell Sciences, Faculty of Advanced Technologies in Medicine, Mazandaran University of Medical Sciences, PO Box: 48175/861, Sari, Iran; 7 Molecular and Cell Biology Research Center, Mazandaran University of Medical Sciences, Sari, Iran; 8 Department of Pharmaceutics, Faculty of Pharmacy, Mazandaran University of Medical Sciences, Sari, 17th Kilometer of Sea Street, PO Box: 48175/861, Sari, Iran

**Keywords:** Dental pulp stem cells, Rosuvastatin, Hyaluronan, Niosomes, Box-Behnken design, Osteogenic differentiation, Bone tissue engineering

## Abstract

Bone tissue engineering necessitates a stem cell source capable of osteoblast differentiation and mineralized matrix production. Dental pulp stem cells (DPSCs), a subtype of mesenchymal stem cells from human teeth, present such potential but face challenges in osteogenic differentiation. This research introduces an innovative approach to bolster DPSCs’ osteogenic potential using niosomal and hyaluronan modified niosomal systems enriched with rosuvastatin. While rosuvastatin fosters bone formation by regulating bone morphogenetic proteins and osteoblasts, its solubility, permeability, and bioavailability constraints hinder its bone regeneration application. Using a Box-Behnken design, optimal formulation parameters were ascertained. Both niosomes were analyzed for size, polydispersity, zeta potential, and other parameters. They displayed average sizes under 275 nm and entrapment efficiencies exceeding 62%. Notably, niosomes boosted DPSCs’ cell viability and osteogenic marker expression, suggesting enhanced differentiation and bone formation. Conclusively, the study underscores the potential of both niosomal systems in ameliorating DPSCs’ osteogenic differentiation, offering a promising avenue for bone tissue engineering and regeneration.

**Graphical Abstract:**

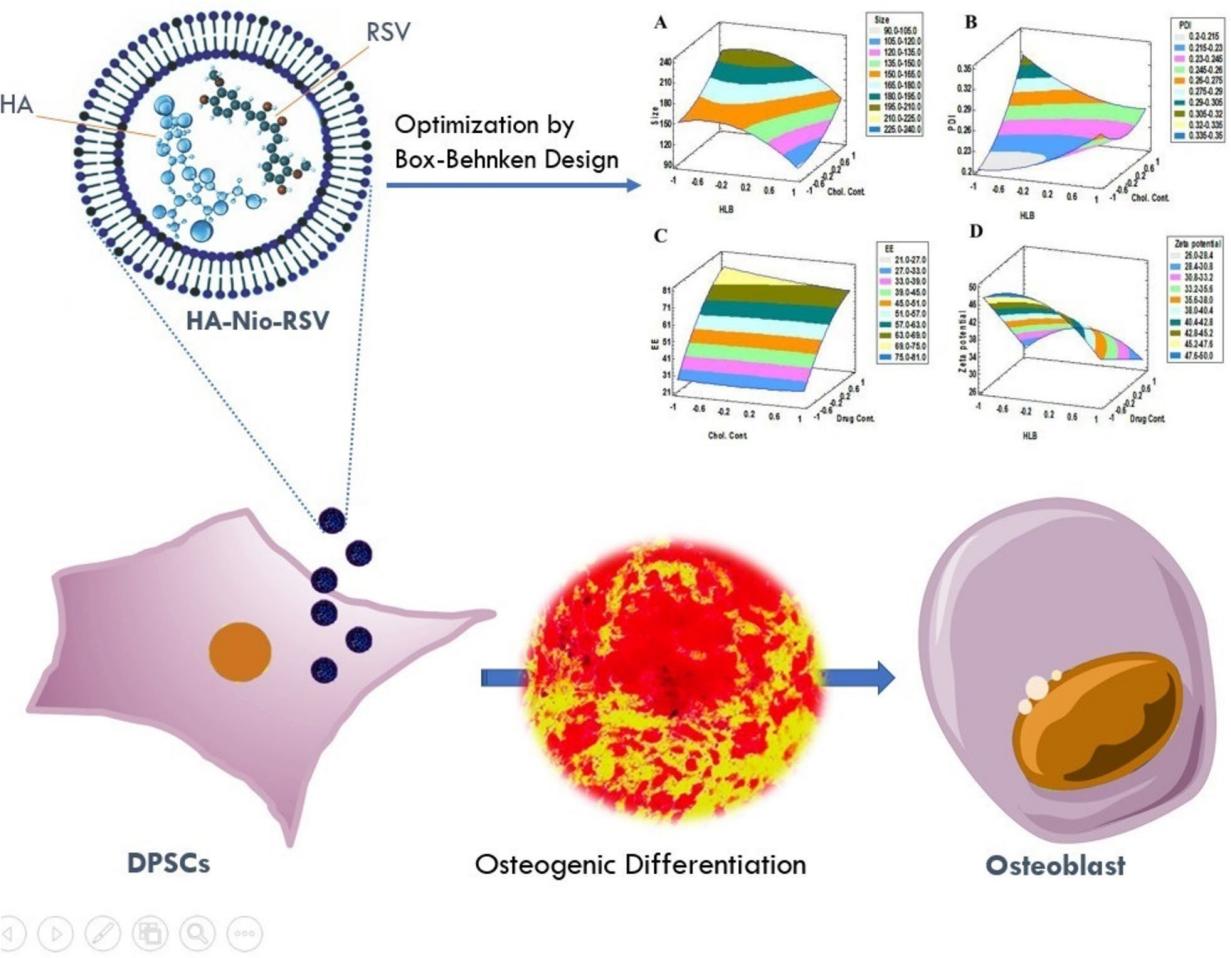

## Introduction

 Dental pulp stem cells (DPSCs) are a type of mesenchymal stem cells that can be isolated from the dental pulp tissue of human teeth. DPSCs have the potential to differentiate into various cell types such as osteoblasts, chondrocytes, adipocytes, and neurons [1]. For this reason, DPSCs are considered a promising source of stem cells for tissue engineering and regenerative medicine, especially for bone and dental applications [2].

However, the osteogenic differentiation and mineralization of DPSCs are often influenced by various factors, such as the culture conditions, the growth factors, and the biomaterials used. Among these factors, biomaterials play a crucial role in providing physical and chemical cues that modulate the behavior and function of DPSCs. Therefore, there is a need to develop novel biomaterials that can enhance the osteogenic potential of DPSCs and improve their therapeutic outcomes [2].

Rosuvastatin (RSV) is a drug that belongs to the class of statins which are widely used to lower cholesterol levels and prevent cardiovascular diseases. RSV has also been reported to have beneficial effects on bone metabolism and osteogenesis by stimulating the expression of bone morphogenetic proteins (BMPs), inhibiting the activity of osteoclasts, and enhancing the activity of osteoblasts. However, RSV has some limitations that hinder its clinical application for bone regeneration, such as its low solubility, low permeability, low bioavailability, and high cytotoxicity. Therefore, there is a need to enhance the delivery and efficacy of RSV using innovative carriers that can overcome these limitations [3].

Niosomes are nanosized vesicles that are composed of nonionic surfactants, cholesterol, and other additives. Niosomes can encapsulate hydrophilic and hydrophobic drugs and deliver them to specific sites in a controlled manner. Niosomes have several advantages over other nanocarriers, such as liposomes and polymeric nanoparticles, such as low cost, high stability, easy preparation, and tunable properties. Therefore, niosomes can be used as an effective delivery system for RSV that can increase its solubility, permeability, bioavailability, and osteogenic effects [4–7].

Hyaluronan (HA) hydrogels have been considered as biocompatible and safe scaffold supports for human dental cell therapies. Therefore, HA can be used as a biocompatible and bioactive additive for niosomes that can improve their physicochemical characteristics, cellular uptake, and biocompatibility. In fact, HA can be used to prepare a new generation of niosomes as a polymeric modified niosomes [4].

Dental pulp stem cells (DPSCs) show great promise as a cell source for a wide range of regenerative medicine uses. The benefits of dental pulp stem cells over mesenchymal stem cells include easier accessibility as they can be obtained from extracted teeth, rapid proliferation capacity, immune modulatory effects [8, 9]. Generally, the DPSCs had more growth rates, less cellular aging, and improve osteogenesis differentiation compared to those of non-dental MSCs cultured [10].

In this article, we aim to develop a niosomal and a novel vesicular HA modified niosomal system loaded with RSV (Nio-RSV and HA-Nio-RSV respectively) and investigate their impact on the osteogenic differentiation of DPSCs. We use the box-Behnken design to optimize the formulation parameters of Nio-RSV and evaluate its physicochemical properties using various techniques. We also assess the cytocompatibility and osteogenic potential of Nio-RSV and HA-Nio-RSV in vitro using DPSCs derived from human dental pulp tissue. We hypothesize that both Nio-RSV and HA-Nio-RSV can stimulate the expression of osteogenic markers and promote the mineralization of DPSCs by modulating various signaling pathways. We expect that our findings will provide new insights into the role of Nio-RSV and HA-Nio-RSV in enhancing the osteogenic differentiation of DPSCs and improving their efficiency for bone tissue engineering and regeneration. To achieve this, several experimental techniques were employed including real-time PCR, Alizarin Red staining, and MTT assay.

## Materials and methods

### Materials

The following materials were used: Rosuvastatin (sipla, india), cholestrol (merck, Germany), span20 (merck, Germany), span40 (merck,Germany), span60 (merc,Germany), Sodium hyaluronate (contipro, Czech Republic), Chloroform, Ethanol and all other chemicals and solvents (merck,Germany). Dental pulp stem cells (Bonyakhte,Iran), β-glycerolphosphate (Merck,Germany), ascorbic acid biphosphate (Sigma‐Aldrich,USA) and dexamethasone (Sigma‐Aldrich, USA), Dulbecco’s modified Eagle’s medium (DMEM), 10% fetal bovine serum (FBS), penicillin/streptomycin, β‐glycerolphosphate (Merck), ascorbic acid biphosphate (Sigma‐Aldrich), dexamethasone (Sigma‐Aldrich).

## Methods

### Preparation of niosomes

Thin film hydration method was used to prepare rosuvastatin niosomes (LABOROTA 4000-efficient, Heidolph, Schwabach, Germany). According to Table 1, a specified quantity of drug, cholesterol, and surfactant was weight and dissolved in specific amounts of solvents (chloroform and ethanol) in a flask. Then, the solvent was evaporated and thin film was prepared on the wall of the flask using rotary evaporator with a specific condition (60 °C ± 2 °C, 150 rpm, 30 min). Then, the thin film layer was hydrated with distilled water. The flask was rotated at 270 rpm, 60 °C ± 2 °C, without vacuum during hydration for 30 min [11]. Thereafter, probe sonicator (bandelin electronic type GM 3100, Berlin, Germany) was used to uniform niosomal dispersion. Sonication condition included 4 cycles of sonication (60 s on − 60 s off). Ultracentrifuge method with 35,329×g force for 60 min [4, 5, 12, 13] was used to purify niosomes using ultracentrifuge (Sigma, Germany).

**Table 1:** Physicochemical characteristics of rosuvastatin niosomes

Run	A HLB of surfactant	B cholesterol concentration	C Drug content	Response 1 Mean vesicle size (nm)	Response 2 PDI (value)	Response 3 Zeta potential (mV)	Response 4 Entrapment Efficiency (%)
F1	1	-1	0	93.27 ± 9.43	0.27 ± 0.01	-34.70 ± 720	68.12 ± 3.75
F2	0	0	0	168 ± 8.94	0.23 ± 0.05	-37.47 ± 1.11	58.82 ± 1.63
F3	-1	0	-1	166.43 ± 7.28	0.23 ± 0.02	-48.53 ± 2.50	21.39 ± 4.95
F4	1	1	0	148.53 ± 13.59	0.23 ± 0.00	-26.23 ± 2.32	48.1 ± 5.8
F5	0	1	-1	214.57 ± 13.02	0.28 ± 0.03	-44.03 ± 5.92	28.3 ± 11.29
F6	0	-1	-1	145.47 ± 10.40	0.26 ± 0.08	-46.03 ± 13.60	25.52 ± 17.15
F7	1	0	1	131.70 ± 3.39	0.28 ± 0.02	-27.97 ± 0.23	65.58 ± 4.28
F8	0	-1	1	164.57 ± 9.31	0.21 ± 0.00	-30.27 ± 1.04	74.83 ± 1.88
F9	-1	0	1	150.30 ± 19.11	0.27 ± 0.03	-28.47 ± 0.50	67.32 ± 2.35
F10	0	0	0	164.23 ± 4.40	0.22 ± 0.03	-43 ± 1.10	48.24 ± 2.43
F11	-1	-1	0	157.10 ± 7.16	0.23 ± 0.01	-37.90 ± 1.78	59.72 ± 3.46
F12	1	0	-1	117.47 ± 14.88	0.29 ± 0.01	-35.43 ± 3.02	32.71 ± 7.08
F13	0	1	1	191.20 ± 7.97	0.34 ± 0.05	-32.20 ± 2.00	70.67 ± 1.35
F14	-1	1	0	214.33 ± 1.66	0.32 ± 0.02	-30.40 ± 2.217	56.22 ± 2.68
F15	0	0	0	164.47 ± 2.39	0.22 ± 0.01	-42.07 ± 2.27	60.77 ± 1.83

### Size, poly dispersity index (PDI) and zeta potential evaluation

Dynamic light scattering (DLS) method (Malvern, Worcestershire, UK) was used to analyze the mean vesicle size and poly dispersity index of the niosome dispersion. Zeta sizer (Malvern, Worcestershire, UK) was also used to analyze the value of zeta potential. To analyze the size and zeta potential of the niosomes, samples were prepared by diluting specific amounts of niosomal dispersion with specific amounts of purified water (1:2) [12, 14].

### Entrapment Efficiency (EE % w/w)

Entrapment efficiency was calculated using the following equation:

$$EE\;\left(\%w/w\right)\;\;=\;\frac{Amount\;of\;the\;rousvastatin\;entrapped\;in\;the\;vesicles}{Total\;amounts\;of\;the\;rousvastatin}\;\times\;100$$

The amounts of rosuvastatin entrapped in the niosomal vesicles were calculated indirectly by subtracting free amounts of rosuvastatin from the initial amounts of rosuvastatin that were used to prepare niosome dispersion. The spectrophotometric method (Jasco, v630, spectrophotometer, Japan) at 244 nm was used to measure the amount of rosuvastatin [4, 5, 12, 13].

### Box-Behnken analysis and preparing optimum formulation with and without hyaluronan

Three factors including HLB of surfactant, cholesterol concentration and drug content with three levels were selected to be studied. Moreover, four responses including mean vesicle size, PDI, zeta potential and entrapment efficiency were considered to be analyzed to determine the effect of each factor on them. Primary data including size, PDI, zeta potential and entrapment efficiency were analyzed mathematically and the optimum formulation was predicted by Box-Behnken design. The formulation with the lowest vesicle size and polydispersity index value and highest values of zeta potential and entrapment efficiency was selected as an optimum formulation. The optimum formulation was prepared and its properties compare with predicted amounts. Another formulation was prepared using hyaluronan gel instead of water as a hydration medium. Hyaluronan gel was first prepared by hydrating a specific amount of hyaluronan in a beaker containing water at room temperature, overnight. Magnetic stirrer (MR 3001, Heidolph, Germany) was used to hydrate hyaluronan polymer in the water. Then optimum formulation was prepared by hyaluronan gel as hydrophilic phase to hydrate the thin film layer. These two formulations (optimum formulation and optimum formulation hydrated by hyaluronan gel) were used for further studies. Mean vesicle size, poly dispersity index (PDI), zeta potential and entrapment efficiency were studied by the methods mentioned previously [4].

### Scanning Electron Microscopy (SEM)

Surface morphology and shape of the rosuvastatin entrapped niosomes and rosuvastatin entrapped niosomes containing hyaluronan were analyzed by SEM. Samples were coated with gold and scanned by the apparatus. Briefly, niosomes were purified by centrifugation method as described previously. Precipitated niosomes were then dried; after, freezing the washed precipitated niosomes at -70 °C, and freeze-drying for 24 h using freeze dryer (CHRIST alpha 1–2, Germany). The dried niosomes were coated with gold and examined using scanning electron microscopy (SNE4500M, USA) [13, 15, 16].

### Transmission Electron Microscopy (TEM)

Lamellarity and shape of the rosuvastatin entrapped niosomes and rosuvastatin entrapped niosomes containing hyaluronan were analyzed by TEM (ZEISS, Germany). Samples were dried on the copper–gold carbon grid and mounted in the instrument to take different photographs.

### Differential scanning calorimetry analysis (DSC)

Dry powder of niosomes was used for DSC analysis. Freeze dryer (alpha 1–2 ld plus, Martin Christ GmbH, Germany) was used to dry niosomal dispersion and make a dry powder from the dispersion. The sizes of the samples were 5 mg that were sealed hermetically in DSC specific pans. Analysis was performed under nitrogen atmosphere with a heating range and scanning rate of (30 − 300 °C) and 10 °C /min respectively, using thermal analysis (Perkin Elmer, PYRIS 6 DSC INTRACOOLER, Netherland).

### Fourier transforms infrared spectroscopy (FTIR)

Samples for FTIR study were prepared by grinding each powder including pure rosuvastatin calcium, hyaluronan, rosuvastatin entrapped niosomes and rosuvastatin entrapped niosomes containing hyaluronan. Then, each powder was mixed with potassium bromide individually. Finally, each powder mixture was pressed by the apparatus to prepare a disc with 10 mm diameter. The disc was analyzed by Perkin-Elmer Spectrum (Perkin Elmer, PYRIS 6 DSC INTRACOOLER, Netherland). One from 4000 − 400 cm^−1^.

### In vitro release studies

To identify the release profile of rosuvastatin entrapped niosomes as a master formula and rosuvastatin entrapped niosomes containing hyaluronan as a master formula containing hyaluronan dialysis method was used and sink condition was established. Sample size was 2 ml and dialysis bag with molecular weight cut off 12,400 from Sigma was used to perform In vitro release study. Dissolution medium was 35 ml phosphate buffer (pH 6.8) and each dialysis bag containing formulation was suspended in it after sealing. Shaker incubator (IKA KS 4000 ic control, Staufen, Germany) was used as an apparatus to perform this study as this apparatus could provide a constant temperature (37 °C) and rotation speed (75 rpm). Spectrophotometric method was used to analyze the amount of rosuvastatin in the dissolution medium at specific intervals. Exact amount of the drawn dissolution medium was replaced by fresh dissolution medium to maintain sink condition [17, 18].

Drug release mechanism study:

Kinetic models including Zero order, Hixon-Crowell, Peppas (Power Law), First order and Higuchi were analyzed to obtain the best kinetic model.

### Stability

A comprehensive stability study was meticulously carried out over a period of 90 days at room temperature to thoroughly investigate and evaluate the stability of the niosomes dispersion. Various characteristic properties, such as mean vesicle size, polydispersity index, and zeta potential, were measured at specific intervals to analyze the stability of the dispersion. By regularly measuring and monitoring these fundamental properties, this study aimed to provide a detailed understanding of the stability profile of the niosomes dispersion.

### DPSCs culture and osteogenic differentiation

Dental pulp stem cells were cultured in Dulbecco’s modified Eagle’s medium (DMEM), 10% fetal bovine serum (FBS) and 1% penicillin/streptomycin. Cells were subcultured every 2 to 3 days by trypsinization as they reached approximately 80% confluency. All cell culture reagents were obtained from Gibco. The cells were subcultured every 2–3 days until they reached the confluency of approximately 80%. Then stem cells were trypsinated and 120,000‒150,000 cells were added to each well of 24 well. The culture medium is changed and osteogenic differentiation medium containing 10 mM β-glycerolphosphate, 50 mg/ml ascorbic acid biphosphate and 100 nM dexamethasone was added for 2 weeks [19, 20]. To induce osteogenic differentiation rosuvastatin was added alone or in combination with niosome as rosuvastatin or rosuvastatin-niosome group respectively. The concentration of rosuvastatin is 1 µmol/l and the control group is without any treatment [8].

### MTT assay

To assess cell viability, MTT (3-(4, 5-dimethylthiazolyl-2)-2, 5-diphenyltetrazolium bromide) assay was done in rosuvastatin, rosuvastatin-niosome, control and blank niosome groups. Cells were cultured for 24houres at 37 °C with 5% CO_2_ in a 96-well plate, with each well containing 10^5^ cells/ well. They were then treated for 5 days. Subsequently, MTT assay was performed following the instructions provided in the kit (Sigma, MTT concentration is 0.5 mg/ml), and the absorbance of the solution was determined by photospectrometry at 570 nm [21].

### RNA extraction and quantitative real-time PCR

Total RNA was isolated from cells and tissues using Qiazol (Qiagen) according to the manufacturer’s protocol. 2 µg total RNA was reverse-transcribed to cDNA using M-MuLV reverse transcriptase (Fermentas, Waltham, Massachusetts, USA) and random hexamer primers. Quantitative real-time PCR assay was done with the ‘SYBR Green I Master Mix kit (Roche, Diagnostics, Penzberg, Germany) by the Rotor-Gene 6000 instrument (Corbett, Sydney, Australia). Cycle threshold (Ct) values were computed as explained [22] and Relative Expression Software Tool (REST, 2009) (Qiagen, Hilden, Germany) was used for determination of fold changes. Expression levels of the target genes were normalized to the expression of GAPDH gene, and relative expression was computed using 2 ^–ΔΔCt^ method [23]. Sequences of primers are prepared in Table 2.

**Table 2:** Sequences of primers used in qPCR

Gene	Sequence 5’-3’
BMP2	F: GCTGTGATGCGGTGGACTGC
	R: CGCTGTTTGTGTTTGGCTTGA
OCN	F: CCCATTGGCGAGTTTGAGAAG
	R: CAAGGCCCGATGTAGTCCAG
RUNX2	F: CTCACTGCCTCTCACTTGCC
	R: CTGTACACACATCTCCTCCCTTC
ALP	F: GGGGACATGCAGTATGAATT
	R: GGCCTGGTAGTTGTTGTGAG
GAPDH	F: CAAGATCATCAGCAATGCCTCC
	R: GCCATCACGCCACAGTTTCC

### Quantification of the ALP activity

The alkaline phosphatase (ALP) activity of DPSCs was analyzed by ALP kit (Millipore) according to the manufacturer’s instructions. Briefly, 5 × 10^4^ DPSCs were seeded into 12-well plates, 21 days after differentiation of DPSCs the alkaline phosphatase activity was analyzed by reading absorbance at 405 nm.

### Alizarin red

Alizarin red test was performed to evaluate the effect of rosuvastatin and rosuvastatin- niosome on bone differentiation. Alizarin red staining indicated the osteoblast biological activity in DPSCs after 21 days of differentiation. To perform the staining, cells were fixed with 4% formaldehyde (m/v) for 35 min, followed by washing with deionized water. Subsequently, the cells were stained with 0.5% Alizarin Red S solution for 40 min at room temperature. After staining, the cells were rinsed with water and observed using an inverted microscope. Quantification of the total mineralized cells was achieved through a distaining procedure involving the addition of 2 ml of cetylpyridinium chloride buffer (10%, w/v) in 10 mM sodium phosphate (pH 7) for 20 min at 37 °C. The concentrations of Alizarin Red S were then determined by measuring absorbance at 570 nm using a microplate reader and referencing an Alizarin Red standard curve in a similar solution. The shift in cell color to red was correlated with their osteogenic activity. This method allowed for the assessment of the osteoblast function and mineralization potential influenced by the presence of rosuvastatin and rosuvastatin-niosomes.

### Statistical analysis

Statgraphic centurion C18 was used to design Box-Behnken statistical model and perform its primary statistical. Other statistical analysis was performed using one-way analysis of variance (ANOVA) followed by Tukey’s post hoc by SPSS Inc. statistical software. *P* value of < 0.05 was considered statistically significant in all statistical analysis.

## Results and discussion

### Formulation optimization using Box-Behnken design

In this study, we explored the effects of cholesterol concentration, hydrophilic-lipophilic balance (HLB) of the surfactant, and drug content on various characteristics of niosomes, including vesicle size, polydispersity index, zeta potential values, and entrapment efficiency. By employing a Box-Behnken design with three factors and three levels, we generated 15 primary predicted formulations and conducted a comprehensive analysis. Through statistical analysis, we assessed the individual and combined impacts of these parameters on niosome properties, gaining insights into their relationships. The design allowed us to identify optimal values for each parameter, facilitating the creation of niosomes with desired attributes.

The results obtained through this approach provide valuable information for the development of optimized niosome formulations with desired properties, enhancing their potential for various pharmaceutical and biomedical applications.

### Analysis of niosome characteristics

According to ANOVA test and Box-Behnken design the quadratic model was the best fitted model for all responses. Consequent equations were as follows:

$$\mathrm{Mean}\;\mathrm{vesicle}\;\mathrm{size}=165.567\;-\;24.6488\mathrm A\;+\;26.0275\mathrm B\;-\;0.77125\mathrm C\;-\;24.8683\mathrm A^{\wedge}2\;-\;0.4925\mathrm{AB}\;+\;7.59\mathrm{AC}\;+\;12.6092\mathrm B^{\wedge}2\;-\;10.6175\mathrm{BC}\;+\;0.776667\mathrm C^{\wedge}2$$

$$\mathrm{PDI}={0.223333\;}+\;0.0025\;\mathrm A\;+\;0.025\mathrm B\;+\;0.005\;\mathrm C\;+\;0.0170833\mathrm{A}^{\wedge}2\;-\;0.0325\mathrm{AB}\;-\;0.0125\mathrm{AC}\;+\;0.0220833\mathrm B^{\wedge}2\;+\;0.0275\mathrm{BC}\;+\;0.0270833\mathrm C^{\wedge}22$$

$$\mathrm{Zetapotential}=40.8467\;-\;2.74625\mathrm A\;-\;2.01\mathrm B\;-\;7.01875\mathrm C\;-\;5.91583\mathrm A^{\wedge}2\;-\;0.2425\mathrm{AB}+2.9\mathrm{AC}\;-\;2.62333\mathrm B^{\wedge}2+0.9925\mathrm{BC}\;-\;0.0808333\mathrm C^{\wedge}$$

$$\mathrm E\;\mathrm{Rosuvastatin}=55.95\;-\;0.2555\mathrm A\;-\;1.6245\mathrm B\;+\;21.311\mathrm C\;-\;1.98125\mathrm A^{\wedge}2\;-\;1.1565\mathrm{AB}\;-\;3.2665\mathrm{AC}+1.10175\mathrm B^{\wedge}2\;-\;1.7345\mathrm{BC}\;-\;7.21475\mathrm C^{\wedge}2$$

**Fig. 1:**
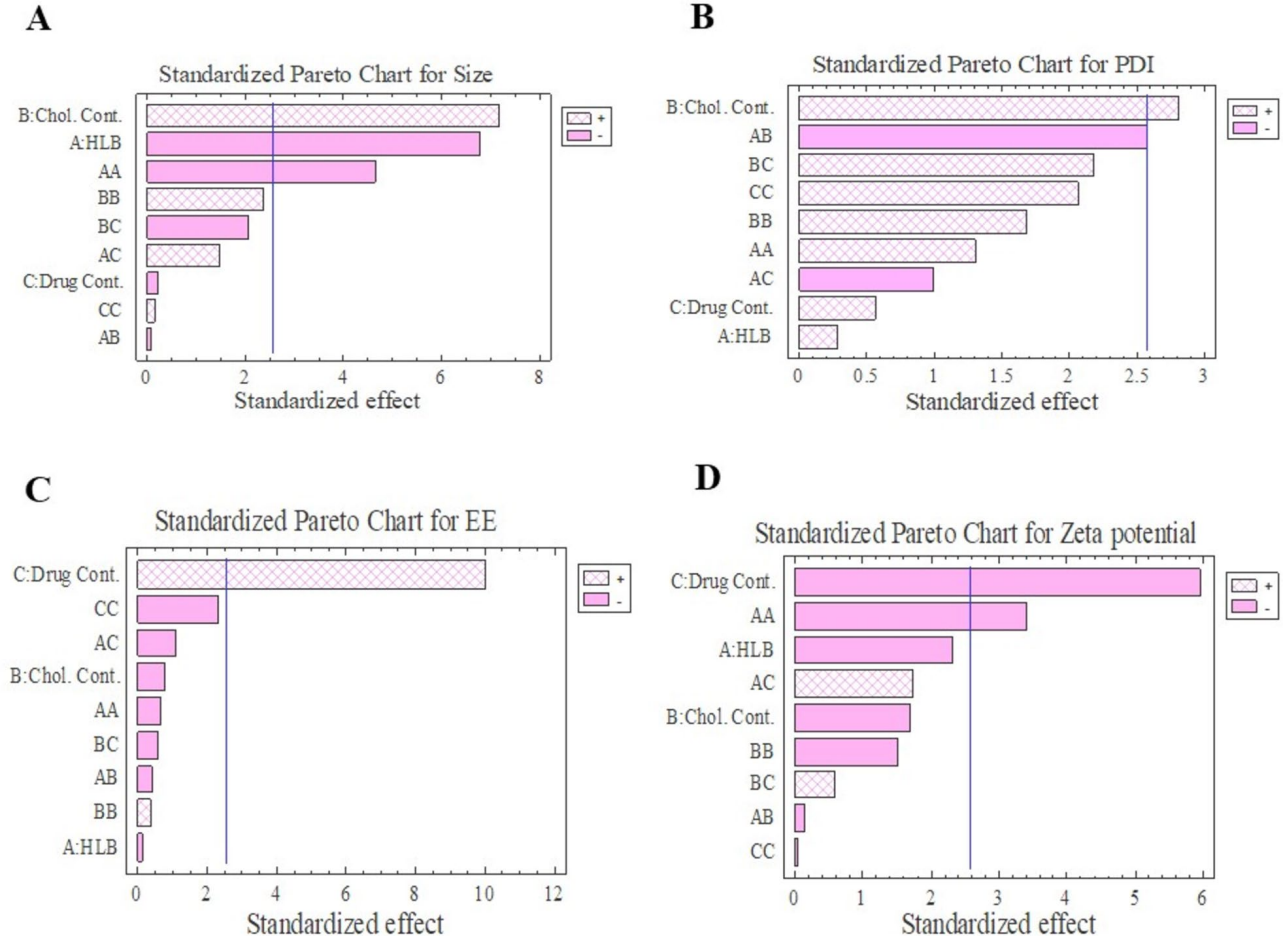
Standardized Pareto chart achieved from anova analysis for 4 responses including Size response, PDI response, Entrapment Efficiency response and Zeta Potential response

**Fig. 2:**
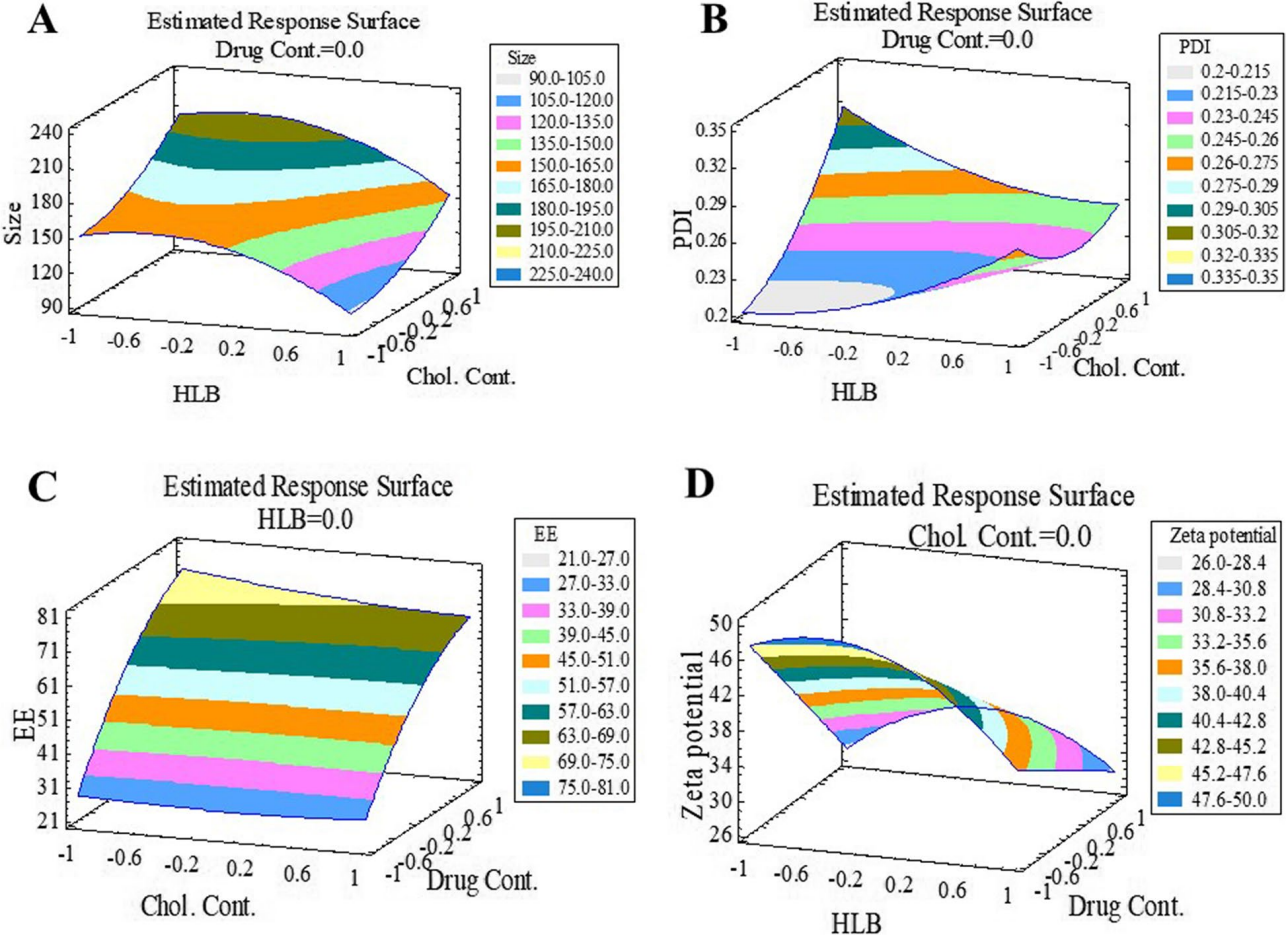
Estimated response surface 3D plots achieved from anova analysis for 4 responses including Size response, PDI response, Entrapment Efficiency response and Zeta Potential response

According to Table 1, DLS method showed that, mean vesicle sizes were in the range of 93 to 214 nm and the optimum formulation mean vesicle size was 165.1 ± 8.07 nm; increasing cholesterol percent caused an increase in the average size of the niosomes from 131.70 nm for F7 to 214.33 nm for F14. This result is supported by the results of ANOVA analysis; briefly, a significant synergistic effect was seen between the mean vesicle size of the niosomes and amount/percent of cholesterol, according to Pareto chart and estimated response surface 3D plot (Figs. 1 and 2). Square root of the cholesterol showed similar results; but, it wasn’t statistically significant. PDI values had significant reverse effect on HLB values (Table 1; Figs. 1 and 2). This significant reverse effect was also seen by the square root of the HLB. Another critical parameter was entrapment efficacy, as shown in Table 1, mean entrapment efficiencies were in the range from 21.39 ± 4.95% to 74.83 ± 1.88%. ANOVA analyses showed, this parameter had a significant direct relation with the amount of drug. In fact, rosuvastatin entrapment efficiency was increased by increasing the amount of the drug. This might be due to the excess amount of the drug that would be present in the lipophilic phase to be entrapped in the bilayers of the niosomes. Reverse relation between entrapment efficiency and HLB value was seen but this effect was not statistically significant. This phenomenon was also seen in the amount of cholesterol (Figs. 1 and 2). Moreover, according to Table 3, rosuvastatin entrapment efficiency of optimum formulation containing hyaluronan was more than optimum formulation without hyaluronan that showed the positive effect of hyaluronan in the entrapment efficiency as previously published [4, 5, 24]. Briefly, incorporating rosuvastatin in the hyaluronan gel and using this gel as a hydration medium might lead to the higher entrapment of rosuvastatin as it can be entrapped in the hydrophilic core; in addition, entrapping in the hydrophobic bilayers. Moreover, hyaluronan might act as a membrane stabilizer and increase bilayers stiffness which leads to less rosuvastatin leaking.

**Table 3:** Physicochemical characteristics of optimum formulation (mean ± SD, *n*=3)

	Predicted data	Observed data	Optimum formulation containing hyaluronan
Size (nm)	151.48	165.1 ± 8.07	272.70 ± 17.05
PDI	0.21	0.25 ± 0.02	0.31 ± 0.03
Zeta Potential (mV)	-39.7	-42.13 ± 1.64	-55.77 ± 2.40
Entrapment Efficiency (%)	60.31	62.49 ± 1.42	73.456 ± 1.26

Polydispersity indexes (PDI) are used to assess dispersion homogeneity. Briefly, low PDI value indicate a homogeny dispersion; while, a high PDI value indicate a heterogenic dispersion with a high range of niosome vesicle size. So, According to the prior publications and results shown in Table 3, niosomes had acceptable homogeneity [25, 26]. According to Table 3; Figs. 1 and 2, PDI values were ranged from 0.21 to 0.34 (mean value 0.26). PDI of the niosomes were increased by increasing the amount of cholesterol from 0.28 to 0.32 for F7 and F14, respectively. According to pareto charts in Fig. 1, HLB value and the amount of the drug also had direct effect on PDI, but this effect was not statistically significant.

Zeta potential is a surface charge of the niosomes and can be used as a parameter to predict niosomes stability. Almost all of the previously published papers agreed with the fact that stable niosome dispersion should have a high amount of zeta potential. Many published papers suggest the range from 20 mv to 30 mv for zeta potential values in a stable system [24, 27]. In this study, the value of zeta potential was in the range from − 26.23 ± 2.32 mv to -48.53 ± 2.5 mv that showed all the prepared formulations had enough value of zeta potential and was stable. That was supported by the stability test results too. Rosuvastatin niosomes were stable after 90 days and there weren’t significant changes in the physicochemical properties of the niosomes. Size, PDI and zeta potential value were 170.21 nm, 0.28 and − 39.43 mv respectively. According to the Figs. 1 and 2, the amounts of zeta potential were increased by decreasing the amounts of drug. A significant reveres effect was also seen between zeta potential and second root of HLB value.

### TEM & SEM analysis

**Fig. 3:**
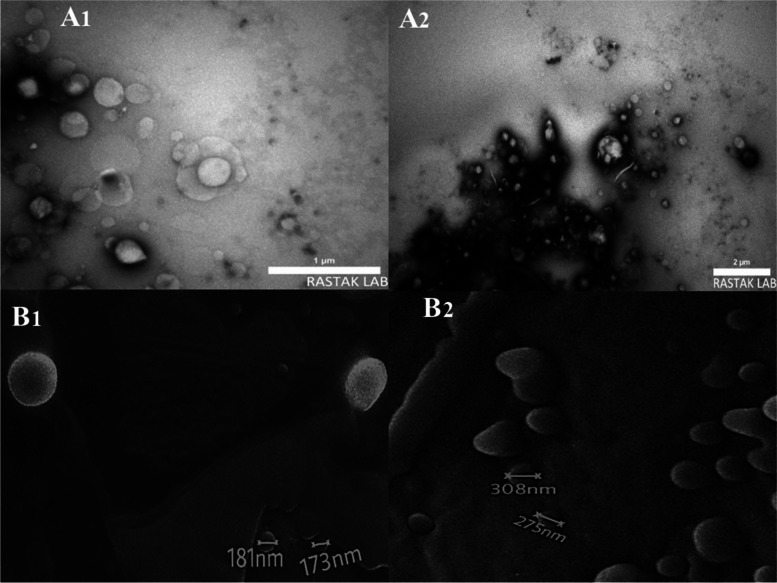
Morphorogical properties of rosuvastatin niosomes and rosuvastatin niosomes containing hyaluronan are showed in TEM and SEM images including TEM images of rosuvastatin niosomes (A1) and rosuvastatin niosomes containing hyaluronan (A2). SEM images of rosuvastatin niosomes (B1) and rosuvastatin niosomes containing hyaluronan (B2)

Figure 3-A_1_ and 3-A_2_ shows TEM images of Nio-RSV and HA-Nio-RSV, respectively. And Figs. 3-B1 and 3-B2 shows SEM images of Nio-RSV and HA-Nio-RSV, respectively. According to Fig. 3 both Nio-RSV and HA-Nio-RSV were spherical. The size of the Nio-RSV in Fig. 3-A_1_ and 3-B_1_ was compatible with the mean vesicle size obtained by DLS method. Similarly, HA-Nio-RSV mean vesicle size obtained by DLS method (Table 3) and SEM analysis in Fig. 3-A_2_ and 3-B_2_ were compatible too.

### Stability analyses

Stability analyses were conducted to assess the physicochemical characteristics of the niosomes over a period of 90 days, and the results demonstrated their long-term stability. The initial sizes of the niosomes were 165.1 ± 8.07 nm, and after 90 days, they maintained a similar size of 170.21 ± 6.1 nm, indicating negligible changes in size over the stability period. The polydispersity index (PDI) value, a measure of particle size distribution, showed a slight increase from 0.25 ± 0.02 to 0.28 ± 0.04, suggesting a minimal change in the uniformity of the niosomes. The zeta potential, which indicates the surface charge of the particles, remained consistent throughout the stability period. The initial value of -42.13 ± 1.64 mV was comparable to the value after 90 days of -39.43 ± 3.82 mV, indicating that the niosomes maintained their surface charge stability. Similarly, the entrapment efficiency, representing the percentage of drug encapsulated within the niosomes, exhibited a stable value of 62.49 ± 1.42% initially and 59.22 ± 3.08% after 90 days, suggesting no significant loss of drug during the stability period. These results demonstrate the robust stability of the niosomes over the 90-day duration, as evidenced by minimal changes in size, uniformity, surface charge, and drug encapsulation efficiency. The consistent values obtained after the stability period indicate that the niosomes maintained their physicochemical characteristics without significant deviations from the initial values. Overall, the stability analyses confirm the long-term stability of the niosomes, supporting their potential as a reliable and durable drug delivery system. The maintained physicochemical properties over the 90-day period highlight the suitability of these niosomes for extended storage and potential applications in pharmaceutical formulations.

### Differential scanning calorimetry (DSC)

**Fig. 4:**
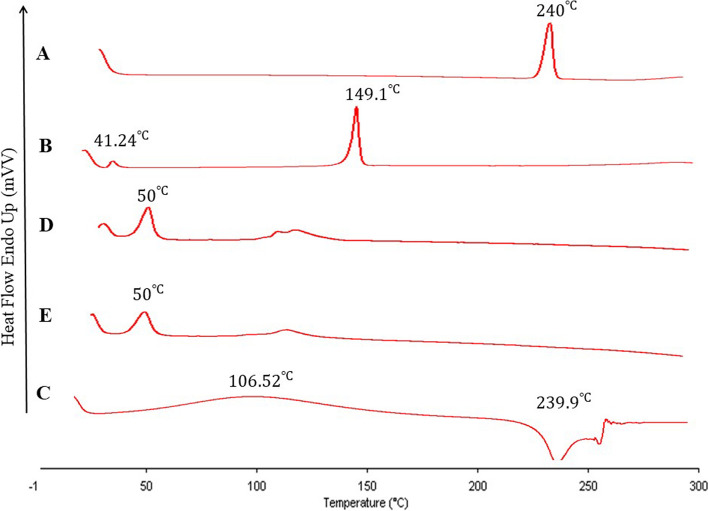
Thermal behavior of different samples are showed in DSC thermograms of each sample including DSC thermograms of pure Rosuvastatin (**A**), Cholestrol (**B**), Hyaluronan (**C**), Rosuvastatin entrapped niosomes (**D**), Rosuvastatin entrapped niosomes containing hyaluronan (**E**)

In Fig. 4, DSC thermograms of pure Rosuvastatin (A), Cholesterol (B), Hyaluronan (C), Rosuvastatin niosomes (D), and Rosuvastatin niosomes containing Hyaluronan (E) are presented. The DSC thermogram of pure Rosuvastatin (Fig. 4-A) exhibits a sharp endothermic peak at 240 °C, which can be attributed to the melting point of Rosuvastatin. This sharp peak is indicative of the crystalline nature of the drug. The thermogram for Cholesterol (Fig. 4-B) shows characteristic endothermic peaks at 41.24 and 149.1 °C. The latter peak is closely aligned with the melting point of Cholesterol. In the thermogram of Hyaluronan (Fig. 4-C), a broad endothermic peak at 106.52 °C is observed, which may represent the polymer’s melting behavior. The presence of a narrow, sharp exothermic peak at 239.9 °C suggests the thermal degradation or destruction of the polymer, corroborating findings reported in the literature. For the Rosuvastatin niosomes (Fig. 4-D) and the formulation containing Hyaluronan (Fig. 4-E), a similar peak around 50 °C is noted in both thermograms. This peak’s consistency across the formulations indicates a reliable attribute of the niosomal preparation, potentially related to the Tg or a phase transition of the niosomal components. Notably, the characteristic sharp endothermic peak of pure Rosuvastatin at 240 °C is absent in the thermograms of the niosome formulations (Fig. 4-D and 4-E), suggesting that the encapsulation within niosomes and the presence of Hyaluronan might alter the crystalline structure of Rosuvastatin, leading to an amorphous form. This alteration could be conducive to the drug’s stability and suggests an interaction between the drug and the niosomal components. Similarly, the characteristic peaks of Cholesterol and Hyaluronan are not distinctly present in the niosome thermograms, implying interactions between these components within the niosome structure. These interactions may influence the stability and encapsulation efficiency of the niosomes, as supported by analogous results in previously published papers [4, 13]. Overall, the DSC thermograms indicate that the niosome formulations, both with and without Hyaluronan, may enhance Rosuvastatin’s stability by altering its physical state.

### FT-IR analysis

**Fig. 5:**
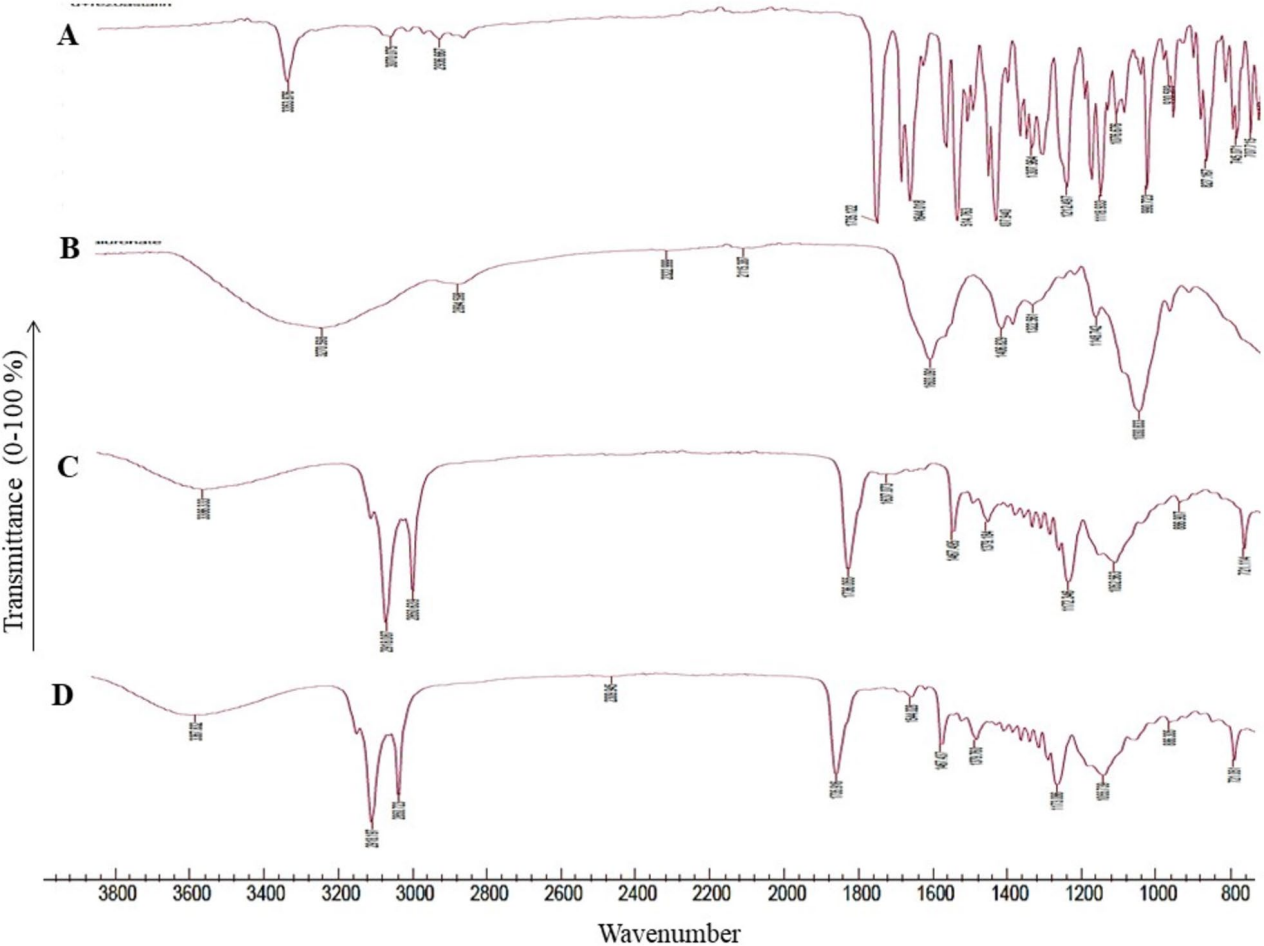
The FT-IR spectra of pure Rosuvastatin (**A**), The FT-IR spectra of hyaluronan (**B**), The FT-IR spectra of Rosuvastatin niosomes (**C**), The FT-IR spectra of Rosuvastatin niosomes containing hyaluronan (**D**) were analyzed to detect possible compatibilities or incompatibilities

According to Fig. 5, Characteristics peaks and identical spectra of rosuvastatin calcium including N-H stretching characteristics peak (2968.55 cm-1), C = O stretching characteristics peak (1732.13 cm − 1), C = C stretching characteristics peak (1546.96 cm-1), =C-H stretching characteristics peak (2922.25 cm-1), N-H bending characteristics peak (3387.11 cm-1), and symmetric bending vibration of CH3 group characteristics peak (1383.01 cm-1) were seen from pure powder (Fig. 5-A) and rosuvastatin entrapped niosomes and hyaluronan containing rosuvastatin entrapped niosomes (Figs. 5-C, D). hyaluronan was also analyzed in this study to detect the possible incompatibilities. Characteristics peaks of hyaluronan were seen in Fig. 5-B. Results showed that no incompatibilities were seen and no new bands appeared [28, 29].

### In vitro release

**Fig. 6:**
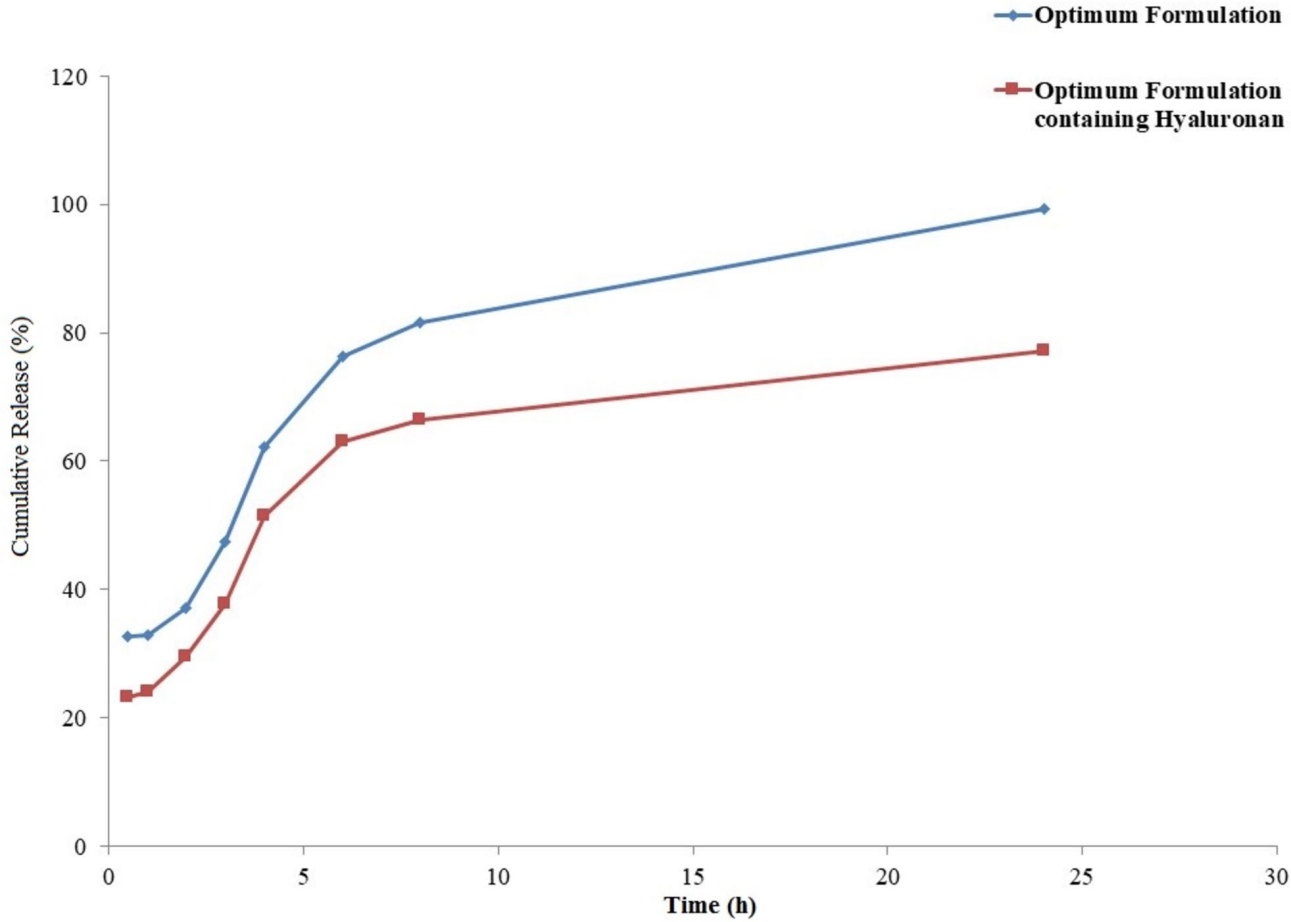
Comparative release profiles of rosuvastatin from optimum formulations with and without hyaluronan over 25 h

According to the data presented in Fig. 6, the in vitro release study revealed distinct release profiles for rosuvastatin from the different formulations. Rosuvastatin entrapped in hyaluronan-containing niosomes exhibited a sustained release pattern, while rosuvastatin entrapped in regular niosomes demonstrated a faster release rate, with complete release occurring within 25 h. The initial burst release observed in the niosomes without hyaluronan could be attributed to the immediate availability of rosuvastatin at the niosome’s interface, it is a common feature for many drug delivery systems intended for immediate therapeutic action. The subsequent sustained release phase, although less pronounced in this formulation, is still beneficial for maintaining drug levels above the minimum effective concentration for a prolonged period. In contrast, the hyaluronan-containing niosomes provided a more moderated release, likely due to the encapsulation of rosuvastatin within the hyaluronan matrix, which creates a barrier to rapid diffusion. This discrepancy in release rates can be attributed to the presence of hyaluronan, which is entrapped within the inner core of the niosomes. The release of rosuvastatin from a gel-like matrix (hyaluronan) within the niosomes is expected to be slower compared to a release from a solution within the niosomes, as observed in previous studies [4, 30]. The release behavior of rosuvastatin from niosomes was analyzed using different mathematical models, and the best fit was achieved with the Higuchi model, yielding an R2 value of 0.965 (as shown in Table 4). The Higuchi model is commonly used to describe the release of a dispersed drug from a homogeneous matrix, where release is controlled by the diffusion of the solvent into the matrix, following Fickian diffusion principles. Moreover, the release behavior of rosuvastatin from hyaluronan-containing niosomes conformed to the Pepas model, which further supports the controlled release mechanism observed in this study. In summary, the in vitro release study demonstrated sustained release characteristics for rosuvastatin from hyaluronan-containing niosomes and faster release kinetics from regular niosomes. The Higuchi model provided the best fit for the release behavior of rosuvastatin from niosomes, indicating diffusion-controlled release from a homogeneous matrix. The observed release profile aligns with previous research and validates the potential of hyaluronan-containing niosomes as a controlled release [5].

**Table 4:** Different kinetic models fitting the release data

**Rosuvastatin entrapped niosomes**						
	**MPE %**	**RSQ**	**K**	**N**	**Slope**	**Intercept**
Zero order	7.60	0.953	0.0627		0.063	0.301
First order	34.16	0.859	0.3674		-0.367	0.296
**Higuchi**	**8.08**	**0.965**	**0.2652**		**0.265**	**0.070**
Peppas (Power Law)	7.42	0.756	0.3515	0.193	0.193	-1.046
Hixon-Crowell	10.76	0.961			0.059	0.044
**Rosuvastatin entrapped niosomes containing hyaluronan**						
	**MPE %**	**RSQ**	**K**	**N**	**Slope**	**Intercept**
Zero order	25.99	0.668	0.0222		0.022	0.331
First order	34.38	0.793	0.0517		-0.052	-0.392
Higuchi	15.98	0.845	0.1451		0.145	0.157
**Peppas (Power Law)**	**11.27**	**0.838**	**0.2643**	**0.363**	**0.363**	**-1.331**
Hixon-Crowell	24.93	0.751			0.013	0.124

### Effect of rosuvastatin on cell viability of DPSCs

The viability of DPSCs upon treatment with various agents is central to ensuring that any therapeutic or investigative substance does not inadvertently compromise cell health. The MTT assay, a widely recognized method for gauging cell viability, was employed to shed light on how DPSCs responded to treatments from control, blank niosome, rosuvastatin, and rosuvastatin-niosome groups.

**Fig. 7:**
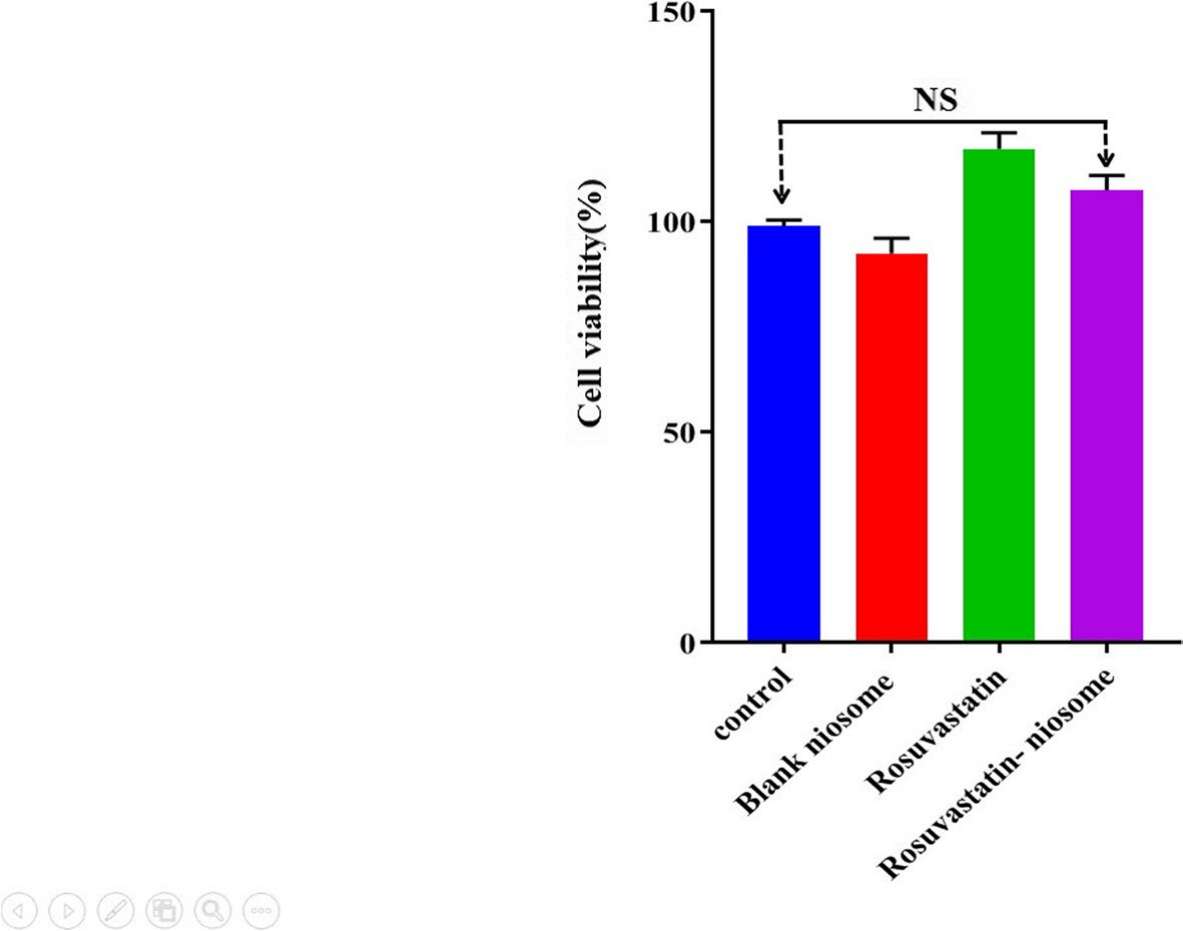
Effect of rosuvastatin and rosuvastatin- niosome on cell viability of DPSCs

From the data illustrated in Fig. 7, it is evident that following a 5 day treatment window, the cell viability percentages across the different groups presented some striking insights. Most notably, the viability values for cells treated with rosuvastatin and rosuvastatin-niosome closely paralleled those from the control and blank niosome groups. This congruence in viability metrics serves as a robust testament to the biocompatibility of rosuvastatin and its niosomal form. Essentially, neither rosuvastatin nor its niosome-combined form demonstrated any discernible cytotoxic effects on DPSCs.

Such findings, besides reinforcing the safety profile of rosuvastatin in the context of DPSCs, also underline the potential of its niosomal delivery system. The fact that rosuvastatin-niosome maintains similar viability percentages as the control and blank niosome groups points to the non-toxic nature of the niosomal system itself. The implication here is profound it suggests that the niosomal delivery doesn’t compromise the inherent safety of the drug, while possibly offering other therapeutic advantages, as alluded to in previous experiments.

### Evaluation of the alkaline phosphatase activity

In the context of assessing early markers of osteogenesis, alkaline phosphatase (ALP) activity assumes paramount significance. It is customarily regarded as an initial indicator of the transition into osteoblast differentiation and, consequently, the commencement of bone matrix mineralization. Guided by this understanding, the present study ventured to elucidate the effect of both rosuvastatin and its niosomal combination on the initiation of the differentiation program in DPSCs.

**Fig. 8:**
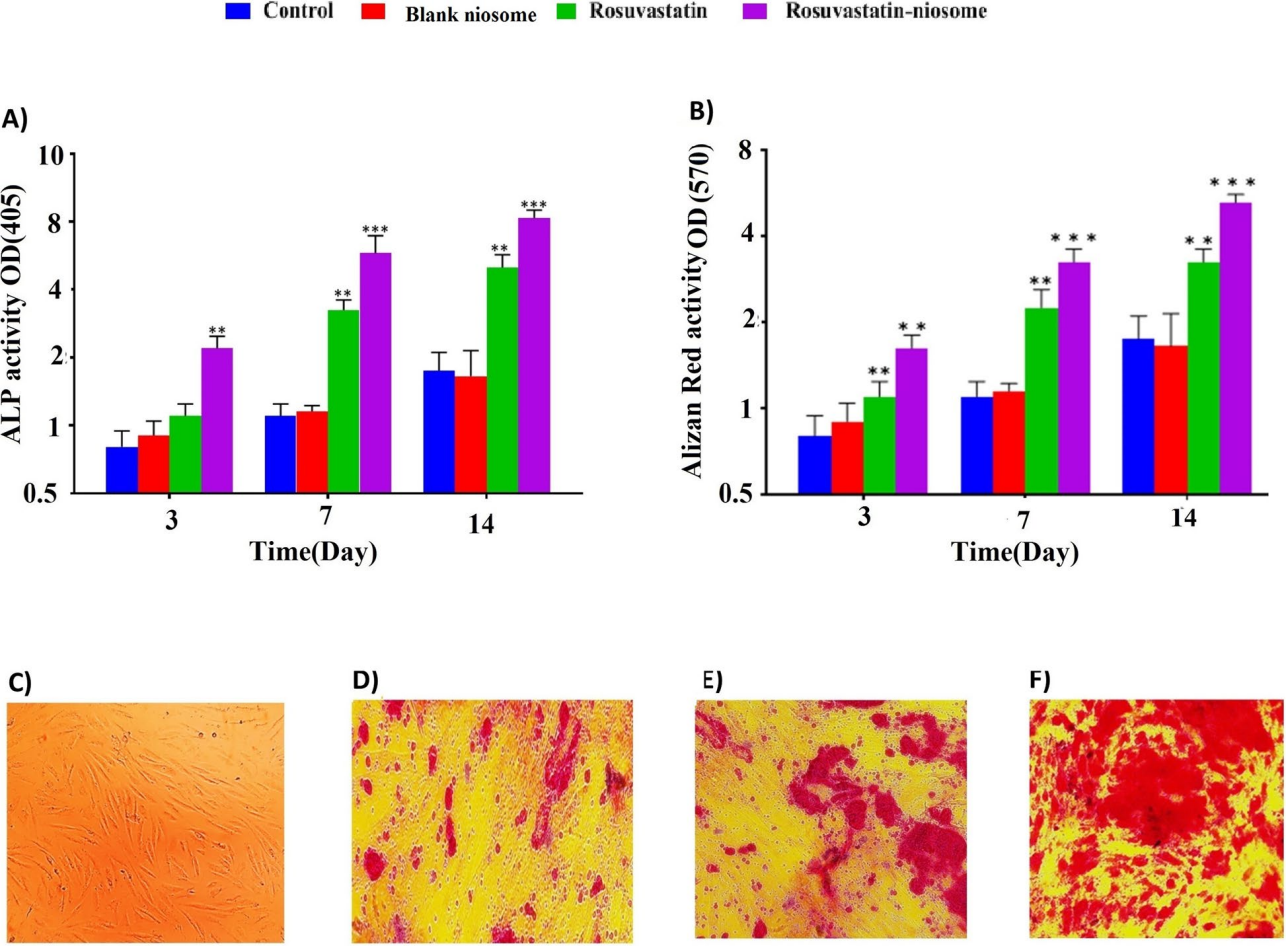
Alkaline phosphatase and Alizarin Red staining assays. ALP activity of DPSCs cultured with, rosuvastatin, rosuvastatin- niosomes and blank niosomes at day 21(8A). The effect of rosuvastatin and rosuvastatin- niosomes treatment on osteogenic differentiation of DPSCs by alizan red staining at day 21. Quantitative Alizarin red staining (8B). Accumulation of calcium deposits in rosuvastatin and rosuvastatin- niosomes groups was shown using Alizarin Red staining. DPSCs (**C**) Control group staining with alizan red (**D**) rosuvastatin group staining with alizan red (**E**) rosuvastatin- niosomes group staining with alizan red (**F**). *n* = 4 (*p < 0.05, **p < 0.01, and ***p < 0.001)

Figure 8A sheds light on the ALP activity across all DPSC groups post their respective treatments. Following a span of 21 days of treatment with either rosuvastatin or the rosuvastatin-niosome, discernible variations were noted in the ALP activity. While an upsurge in ALP activity was observed in the group treated with rosuvastatin, the rosuvastatin-niosome group exhibited a notably elevated activity. This heightened activity was not only significantly above the control group but also markedly outperformed the blank niosome groups after the 21-day period.

Drawing from these observations, it becomes apparent that the niosomal form of rosuvastatin exhibits a pronounced effect in instigating the osteogenic differentiation of DPSCs. The amplified ALP activity in the rosuvastatin-niosome group underscores the potential of niosomes in enhancing the osteogenic capability of the drug. It might be inferred that the niosome, acting as a drug delivery system, could be offering a more controlled and sustained release, leading to increased cellular uptake and, consequently, superior osteogenic outcomes.

### Evaluation of the alizarin red activity

The potential osteogenic effects of rosuvastatin, both alone and in a rosuvastatin-niosome combination, were scrutinized through Alizarin red staining [31]. The purpose was to understand the impact of these treatments on bone differentiation in DPSCs.

The obtained results from Fig. 8B-F vividly depict the influence of both treatment modalities on osteoblast function in different DPSC groups. Here, the integral role of osteoblasts in bone formation and their function was marked by the intensity and extent of alizarin red staining. Post a 21-day treatment, distinct variations were observed in the staining patterns between the groups.

Analyzing calcium content, which serves as a fundamental osteogenic marker, offered intriguing insights. While there was a perceptible augmentation in the calcium content in the rosuvastatin group, it was the rosuvastatin-niosome combination that truly stood out. The alizarin red activity in this group was not only significantly higher than the control but also surpassed the results from the blank niosome group after the stipulated 21-day period. This differential staining activity, which is indicative of calcium deposition and osteoblastic activity, underscores the enhanced osteogenic potential of the rosuvastatin-niosome combination.

These outcomes present compelling evidence to suggest that niosomal encapsulation or delivery of rosuvastatin augments its osteogenic efficacy. This enhancement might be attributed to improved drug delivery, sustained release, or increased cellular uptake provided by the niosome. In essence, the rosuvastatin-niosomes do not merely promote osteogenesis but appear to also steer the programming fate of DPSCs. The underlying mechanisms warrant further exploration, but these findings pave the way for innovative therapeutic strategies in bone tissue engineering and regeneration.

#### Osteogenic mRNA expressions in DPSCs

**Fig. 9:**
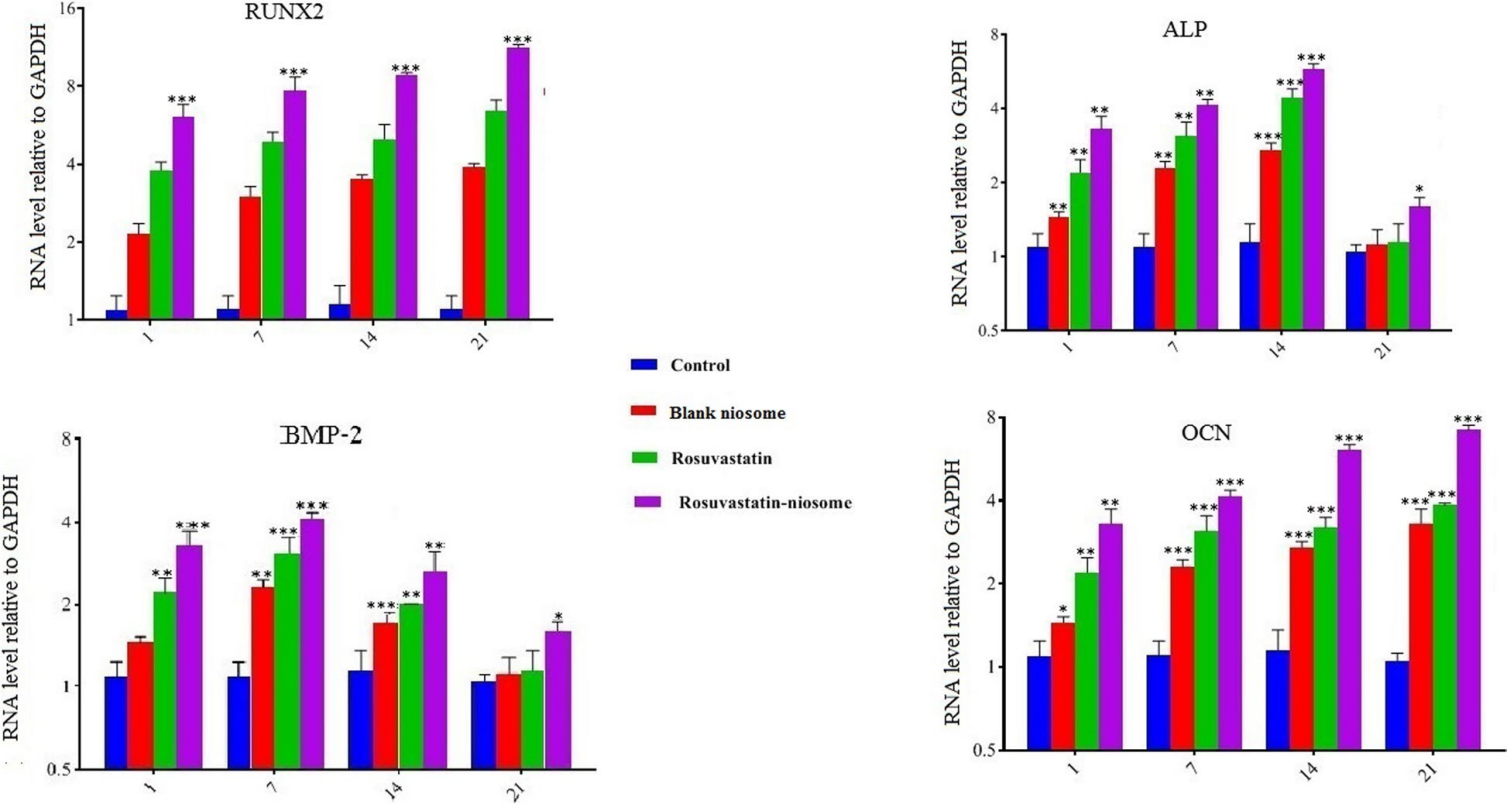
Reverse transcription-quantitative polymerase chain reaction (RT-qPCR) analysis of expression of various osteogenic related markers. Runx2, BMP-2, ALP and OCN were analyzed after exposure of DPSCs to rosuvastatin, rosuvastatin- noisome and blank niosomes at day 1,7,14 and 21. *n* = 4 (*p < 0.05, **p < 0.01, and ***p < 0.001)

The potential of rosuvastatin and rosuvastatin-niosomes in influencing osteogenic differentiation in DPSCs was investigated by examining the expression of vital osteogenic markers. Figure 9 presents the data from quantitative RT-PCR analyses which provide a detailed perspective on the behavior of these compounds when exposed to DPSCs.

Upon analysis, both rosuvastatin and rosuvastatin-niosomes revealed an impressive ability to upregulate the expression of crucial osteogenic-related genes, namely BMP-2, Runx2, ALP, and OCN. Here, OCN characteristically marks the late stages of osteogenesis. In contrast, BMP and ALP emerge as early to intermediate markers, playing pivotal roles during the mineralization phase. Crucially, RUNX2 has a renowned significance in osteoblast development.

Interestingly, the study showcased that the rosuvastatin-niosome setup had a clear edge, with its gene expression levels shooting up considerably higher than its counterparts: rosuvastatin, blank niosomes, and even the control group.

The garnered results shed light on the noteworthy osteogenic potential of both rosuvastatin and its niosome-incorporated variant. While rosuvastatin alone does a commendable job, encapsulating it within niosomes gives it an extra punch. This amplified osteogenic potential, as witnessed by the pronounced upregulation of osteogenic markers, is indicative of the synergy between rosuvastatin and the niosomal delivery system.

The noticeable distinction in gene expression between the rosuvastatin-niosome group and the others accentuates the potential of the niosomal system in enhancing drug delivery and efficacy. This approach seems to foster a more conducive environment for the osteogenic differentiation of DPSCs.

## Conclusion

In conclusion, vesicular systems have emerged as highly beneficial carrier systems for drug delivery. Based on the findings of this study, the incorporation of rosuvastatin in niosomes led to a significant increase in the expression of osteogenesis responsible genes, including BMP-2, Runx2, ALP, and OCN, in dental pulp stem cells (DPSCs), thereby inducing osteogenic differentiation. This highlights the potential of niosomes as a promising vehicle for promoting osteogenesis. Furthermore, the inclusion of hyaluronan in the composition of niosomes demonstrated the ability to modify the properties of conventional niosomes. This modification resulted in niosomes with improved entrapment efficiency and extended release properties. The presence of hyaluronan in the niosomal structure offers opportunities for enhancing the performance and functionality of these vesicular systems, making them even more advantageous for targeted drug delivery applications. Overall, these findings underscore the promising potential of niosomes as carrier systems for drug delivery, particularly in promoting osteogenesis and achieving controlled release of therapeutics. Further exploration and optimization of niosomes, with a focus on incorporating bioactive compounds and tailoring their properties, can pave the way for their wider application in the field of regenerative medicine and targeted therapy.

## Data Availability

The data that support the findings of this study are available from corresponding authors but restrictions apply to the availability of these data, which were used under license for the current study, and so are not publicly available. Data are however available from the authors upon reasonable request and with permission of corresponding authors.
